# Nomogram based on Prognostic Nutritional Index (PNI) for predicting acute radiation proctitis in locally advanced rectal cancer patients with neoadjuvant chemoradiotherapy

**DOI:** 10.7717/peerj.21364

**Published:** 2026-06-02

**Authors:** Yao Wang, Yafang Hong, Ying Wang, Junjian Lin, Hongdan Guan, Zihan Zhou, Benhua Xu

**Affiliations:** 1 Department of Radiation Oncology, Fujian Medical University Union Hospital, Fuzhou, China; 2 Fujian Key Laboratory of Intelligent Imaging and Precision Radiotherapy for Tumors, Fujian Medical University, Fuzhou, China; 3 Clinical Research Center for Radiology and Radiotherapy of Fujian Province (Digestive, Hematological and Breast Malignancies), Fuzhou, China

**Keywords:** Rectal cancer, Radiation proctitis, Prognostic nutritional index (PNI), Nomogram

## Abstract

**Background:**

Radiation-induced proctitis is a frequent adverse effect that negatively impacts the quality of life in rectal cancer patients receiving neoadjuvant chemoradiotherapy (nCRT). Identifying high-risk factors and predicting their occurrence are crucial for preventing acute radiation proctitis (ARP). The aim of this study was to identify potential risk factors and develop a nomogram to predict the risk of ARP.

**Methods:**

A total of 561 locally advanced rectal cancer (LARC) patients from January 1, 2019 to December 31, 2021 were collected. Patients were randomly allocated to the training and validation cohorts in a 7:3 ratio. Univariate and multivariate logistic regression analyses were conducted to determine potential predictive factors. A nomogram was created using predictive factors for ARP. The model’s performance was assessed using the area under the receiver operating characteristic curve (AUC), calibration curves, and decision curve analysis (DCA).

**Results:**

Among 561 patients, 131 were diagnosed with ARP within 90 days after completion of radiotherapy. Multivariable analysis identified Body Mass Index (BMI), tumor volume (TV), and Prognostic Nutritional Index (PNI) as independent predictors for ARP. Their odds ratios (OR) and 95% confidence intervals (95% CI) were as follows: BMI (0.394, 0.230–0.675, *P* < 0.001), TV (2.242, 1.366–3.680, *P* = 0.001), and PNI (0.470, 0.279–0.790, *P* = 0.004). The nomogram showed moderate discriminative ability, with an AUC of 0.689 (95% CI [0.629–0.749]) in the training cohort and 0.725 (95% CI [0.636–0.815]) in the validation cohort. Calibration curves showed acceptable agreement between predicted and observed risks, and DCA suggested potential clinical usefulness within a range of threshold probabilities.

**Conclusion:**

Lower BMI and PNI, and higher TV were independent predictors for ARP in LARC patients receiving nCRT.

## Introduction

Rectal cancers constitute 30–35% of colorectal cancers, with around 50% diagnosed at a locally advanced stage (Sung et al., 2021; Ng, Ngan & Leong, 2022). Neoadjuvant chemoradiation therapy (nCRT) is an effective treatment for patients with locally advanced rectal cancer (LARC) (Adachi et al., 2016). While neoadjuvant pelvic radiotherapy offers advantages, irradiating vital organs poses considerable challenges during treatment (Aghili et al., 2020; Van der Valk et al., 2020).

Acute radiation proctitis (ARP) frequently occurs as a side effect in patients receiving pelvic radiation therapy (Lynn et al., 2024). These patients experience symptoms such as bleeding, diarrhea, pain, and urgency, leading to diminished quality of life, radiotherapy failure, and poor prognosis (Dalsania et al., 2021). Despite advancements in the medical treatments of ARP, therapeutic outcomes remain unsatisfactory. Therefore, the early prevention and diagnosis of ARP are of vital practical significance for rectal cancer treatment.

Nutritional status is crucial in forecasting the efficacy of cancer treatment and associated complications. Body mass index (BMI) is also a widely used indicator of nutritional status (Shi et al., 2022). The Prognostic Nutritional Index (PNI), derived from serum albumin levels and peripheral blood lymphocyte counts, has recently been recognized as an effective measure for assessing the immunonutritional status and predicting the prognosis of cancer patients across various solid tumors (Nogueiro et al., 2022; Oba et al., 2020; Wang et al., 2021a). Critically, the PNI integrates nutritional status and systemic inflammation, both of which play important roles in chemoradiotherapy. Malnutrition and compromised immunity can directly impair tissue repair processes and limit patients’ tolerance to radiation-induced toxicity (Hua et al., 2020; Shi et al., 2021). In addition, a previous study reported that PNI is tightly related to the severe hematologic side effects of radiochemotherapy (Liu et al., 2021b). However, no studies have explored the relationship between PNI and ARP in patients with LARC.

In this study, we analyzed the incidence of ARP in LARC patients undergoing nCRT and collected the clinical features and hematological parameters. We then analyzed the risk factors and first report a novel nomogram model based on PNI to predict the possibility of ARP occurrence in LARC patients, so as to give appropriate intervention measures in advance, reduce the impact of related adverse reactions on patients, improve patient compliance with treatment, thereby enhancing treatment outcomes.

## Materials and Methods

### Patients

In this retrospective study, patients with LARC who underwent nCRT at Fujian Medical University Union Hospital between January 1, 2019, and December 31, 2021 were identified from the institutional database. The inclusion criteria were: (1) all patients were diagnosed with LARC through a combination of pathology and imaging, (2) all patients received nCRT with a total radiation dose of 45.0–50.4 Gy in 25–28 fractions, supplemented by either oral capecitabine alone or 2–6 cycles of the CAPOX/FOLFOX6 regimen, and (3) all patients having a Karnofsky performance score (KPS) of at least 70. Exclusion criteria were: (1) incomplete radiotherapy or short-course radiotherapy, (2) unknown pre-treatment stage, and (3) history of chronic diarrhea or inflammatory bowel diseases. This study was approved by the Institutional Review Board of Fujian Medical University Union Hospital (2025kY445). Written informed consent was obtained from all participants included in this study.

### Data collection

This was a single-center retrospective cohort study. Patients from January 2019 to December 2021 were collected. Clinicopathological features of patients prior to RT included gender, age, BMI, smoking and alcohol history, diabetes, cardiopulmonary diseases, distance from the anal verge, tumor volume (TV), and hematological parameters such as CEA, CA199, NEU, WBC, LYM, HB, and ALB. Patients with missing data in variables required for multivariable analysis were excluded during the initial cohort selection process. A total of 561 eligible patients were included in the study. Missing data were present for some variables used in the descriptive analyses (*e.g.*, CEA and CA199), and no imputation was performed. We included inflammation-related indices like platelet-to-lymphocyte ratio (PLR), neutrophil-to-lymphocyte ratio (NLR), systemic immune-inflammation index (SII), and nutrition-related indices such as PNI and lymphocyte-to-activated neutrophil ratio (LANR). The SII was defined as NLR × platelet count (×10^9^/L) (Hsu et al., 2022). PNI was defined as serum ALB (g/L) +5 × serum LYM count (×10^9^/L) (Zhang et al., 2022). LANR was defined as lymphocyte × ALB/NEU (Liang et al., 2021).

All patients underwent high-resolution pelvic magnetic resonance imaging (MRI) approximately one week prior to nCRT initiation. TV was estimated using the ellipsoid approximation formula *V*=* 0.52* × *L* × *D*^2^. The longest cranial-caudal diameter (L) and the maximum perpendicular diameter (D) were measured using the built-in tools of the PACS system. To ensure reliability, L and D were measured independently by two experienced physicians. The inter-observer agreement was excellent for both L (Intraclass Correlation Coefficient (ICC) = 0.992, 95% CI [0.988–0.995]) and D (ICC = 0.957, 95% CI [0.940–0.971]). The preoperative stage was assessed using the 8th edition of the American Joint Committee on Cancer Staging Manual.

### Evaluation of radiation-induced ARP

ARP was defined as proctitis occurring during nCRT or within ≤90 days after completion of radiotherapy. The clinical severity of ARP was evaluated by documenting bowel movements, stool consistency, rectal bleeding, nocturnal bowel movements, abdominal pain, rectal burning/tenesmus, and a self-assessment of the impact of symptoms on daily activities. Each factor was scored on a scale from 0 (normal) to 3 (severely abnormal), resulting in a maximum overall score of 21 (Vernia et al., 2000). In this study, ARP was defined as a clinical symptom score of 6 or higher. Patients with a clinical score of <6 were categorized into the non-ARP group.

### Nomogram construction and validation

Patients were randomly categorized into training and validation cohort at a 7:3 ratio. Univariate analysis was conducted on clinical characteristics and hematological parameters in the training cohort to investigate their association with ARP. Variables with *P* < 0.05 were placed in the multivariable analysis. Chemotherapy regimen was included as a clinically relevant covariate in the multivariable model. Variables with *P* < 0.05 in multivariable logistic regression were considered independent predictors to develop the nomogram model. The multivariable model’s discrimination was assessed by the area under the receiver-operating characteristic curve (AUC). Multicollinearity was evaluated using tolerance and the variance inflation factor (VIF). The model’s calibration was assessed for goodness-of-fit by employing a calibration curve and the Hosmer-Lemeshow test. To illustrate the utility of the nomogram, decision curve analysis (DCA) was employed to evaluate the net benefits of the model across different threshold probabilities. Model performance was assessed in the validation cohort. In addition, internal validation was performed using bootstrap resampling (500 repetitions) to obtain optimism-corrected estimates of discrimination and calibration according to TRIPOD recommendations.

### Statistical analysis

The analysis utilized IBM SPSS Statistics v26.0, GraphPad Prism v9.0.0, and R v4.2.3 (R Core Team, 2023). A Mann–Whitney U test or an independent-sample, unpaired, two-tailed *t*-test was applied to assess differences in continuous variables. The receiver operating characteristic (ROC) curve was utilized to determine the optimal threshold for continuous variables. The predictive capability of the predictors was evaluated using the AUC. A higher AUC value indicates a stronger predictive ability. A chi-square test or Fisher’s exact test was used to compare categorical variables. Multivariable analysis was conducted to calculate the odds ratio (OR) with a 95% confidence interval (95% CI) and to identify independent predictors of ARP. Differences were considered statistically significant if the two-sided *P* < 0.05.

## Results

### Patient characteristics

**Figure 1:**
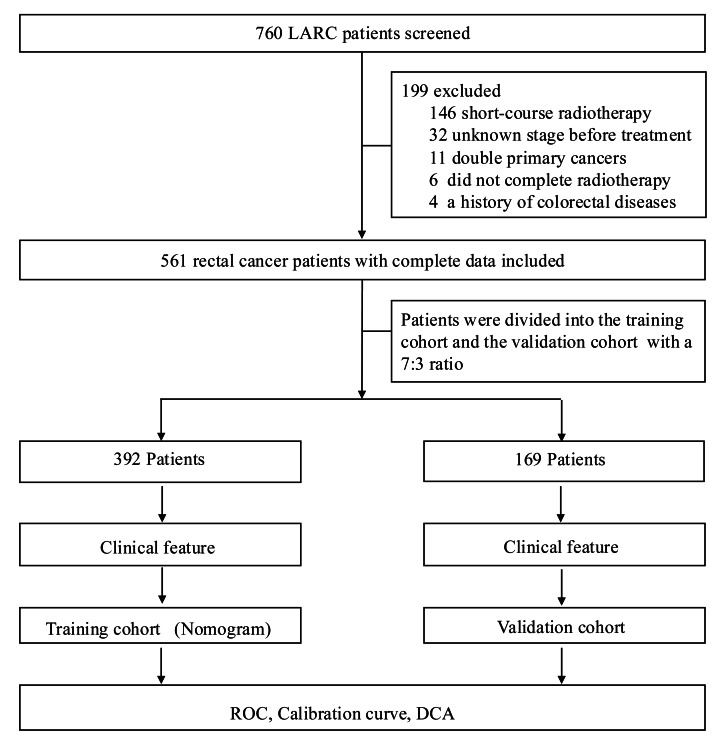
Flowchart of the study. A total of 760 patients with locally advanced rectal cancer (LARC) were screened. A total of 561 patients were eligible for analysis. Patients were randomly assigned to the training cohort (*n* = 392) and the validation cohort (*n* = 169) in a 7:3 ratio. Clinical characteristics were assessed, and the training cohort was used to construct the predictive nomogram.

We retrospectively reviewed 561 eligible patients (Fig. 1). A total of 23.35% of patients suffered from ARP. Of these, the proportion of patients with high TV and CEA value in ARP group were 51.58% and 65.59%, respectively. Furthermore, most patients in the ARP group exhibit lower BMI, ALB, and PNI values compared to those in the non-ARP group (Table 1). The 561 patients were allocated into training cohort and validation cohort. The training cohort included 392 patients, of which 260 were male, with an age average of 57.20 ± 11.09 years. The validation cohort consisted of 169 patients, of which 111 were male, with a mean age of 58.53 ± 11.58 years. The clinical features were displayed in Table 2. There were no significant differences observed between the training and validation cohort, as all *P*-values were greater than 0.05.

**Table 1:** The association between clinical and pathological characteristics and ARP.

Variables	Training cohort (*n* = 392)	No ARP (*n* = 297)	ARP (*n* = 95)	Statistic	*P*
Gender, n (%)				*χ*^2^= 1.000	0.317
Female	132 (33.673)	96 (32.323)	36 (37.895)		
Male	260 (66.327)	201 (67.677)	59 (62.105)		
Age, n (%)				*χ*^2^= 0.835	0.361
<60	211 (53.827)	156 (52.525)	55 (57.895)		
≥60	181 (46.173)	141 (47.475)	40 (42.105)		
BMI, n (%)				*χ*^2^= 15.416	**<**.**001**
<24	225 (57.398)	154 (51.852)	71 (74.737)		
≥24	167 (42.602)	143 (48.148)	24 (25.263)		
Smoking, n (%)				*χ*^2^= 2.905	0.088
No	252 (64.286)	184 (61.953)	68 (71.579)		
Yes	140 (35.714)	113 (38.047)	27 (28.421)		
Alcohol, n (%)				*χ*^2^= 1.203	0.273
No	254 (64.796)	188 (63.300)	66 (69.474)		
Yes	138 (35.204)	109 (36.700)	29 (30.526)		
Diabetes, n (%)				*χ*^2^= 0.101	0.750
No	358 (91.327)	272 (91.582)	86 (90.526)		
Yes	34 (8.673)	25 (8.418)	9 (9.474)		
Cardiopulmonary diseases, n (%)				*χ*^2^= 0.488	0.485
No	312 (79.592)	234 (78.788)	78 (82.105)		
Yes	80 (20.408)	63 (21.212)	17 (17.895)		
Distance from the anal verge, n (%)				*χ*^2^= 0.024	0.877
<5	176 (44.898)	134 (45.118)	42 (44.211)		
≥5	216 (55.102)	163 (54.882)	53 (55.789)		
TV, n (%)				*χ*^2^= 14.237	**<**.**001**
<7.51	253 (64.541)	207 (69.697)	46 (48.421)		
≥7.51	139 (35.459)	90 (30.303)	49 (51.579)		
Chemotherapy regimen				*χ*^2^= 2.42	0.120
capecitabine	163 (41.58)	130 (43.77)	33 (34.74)		
Combination	229 (58.42)	167 (56.23)	62 (65.26)		
Chemotherapy cycles				*χ*^2^= 0.00	1.000
≥3	213 (93.01)	155 (92.81)	58 (93.55)		
<3	16 (6.99)	12 (7.19)	4 (6.45)		
CEA, n (%)				*χ*^2^= 4.661	**0**.**031**
<3.85	167 (44.063)	135 (47.203)	32 (34.409)		
≥3.85	212 (55.937)	151 (52.797)	61 (65.591)		
CA199, n (%)				*χ*^2^= 1.980	0.159
<13.7	200 (54.201)	146 (52.143)	54 (60.674)		
≥13.7	169 (45.799)	134 (47.857)	35 (39.326)		
NEU, n (%)				*χ*^2^= 1.182	0.277
<5.24	333 (84.949)	249 (83.838)	84 (88.421)		
≥5.24	59 (15.051)	48 (16.162)	11 (11.579)		
WBC, n (%)				*χ*^2^= 0.127	0.722
<6.63	254 (64.796)	191 (64.310)	63 (66.316)		
≥6.63	138 (35.204)	106 (35.690)	32 (33.684)		
LYM, n (%)				*χ*^2^= 1.410	0.235
<1.54	149 (38.010)	108 (36.364)	41 (43.158)		
≥1.54	243 (61.990)	189 (63.636)	54 (56.842)		
HB, n (%)				*χ*^2^= 1.261	0.261
<137.5	224 (57.143)	165 (55.556)	59 (62.105)		
≥137.5	168 (42.857)	132 (44.444)	36 (37.895)		
ALB, n (%)				*χ*^2^= 5.720	**0**.**017**
<41.55	161 (41.071)	112 (37.710)	49 (51.579)		
≥41.55	231 (58.929)	185 (62.290)	46 (48.421)		
PLR, n (%)				*χ*^2^= 0.006	0.938
<174.4	139 (35.459)	105 (35.354)	34 (35.789)		
≥174.4	253 (64.541)	192 (64.646)	61 (64.211)		
NLR, n (%)				*χ*^2^= 0.124	0.725
<2.81	282 (71.939)	215 (72.391)	67 (70.526)		
≥2.81	110 (28.061)	82 (27.609)	28 (29.474)		
SII, n (%)				*χ*^2^= 0.011	0.918
<415	118 (30.102)	89 (29.966)	29 (30.526)		
≥415	274 (69.898)	208 (70.034)	66 (69.474)		
PNI, n (%)				*χ*^2^= 11.671	**<**.**001**
<48.08	104 (26.531)	66 (22.222)	38 (40.000)		
≥48.08	288 (73.469)	231 (77.778)	57 (60.000)		
LANR, n (%)				*χ*^2^= 1.441	0.230
<18.67	169 (43.112)	123 (41.414)	46 (48.421)		
≥18.67	223 (56.888)	174 (58.586)	49 (51.579)		

**Notes.**

*χ*^2^ Chi-square test

Bold values indicate statistical significance (*P* < 0.05).

**Table 2:** Patient characteristics.

Variables	Total (*n* = 561)	Training cohort (*n* = 392)	Validation cohort (*n* = 169)	Statistic	*P*
Gender, n (%)				*χ*^2^= 0.022	0.882
Female	190 (33.868)	132 (33.673)	58 (34.320)		
Male	371 (66.132)	260 (66.327)	111 (65.680)		
Age, n (%)				*χ*^2^= 3.310	0.069
<60	316 (56.328)	211 (53.827)	105 (62.130)		
≥60	245 (43.672)	181 (46.173)	64 (37.870)		
BMI, n (%)				*χ*^2^= 1.359	0.244
<24	313 (55.793)	225 (57.398)	88 (52.071)		
≥24	248 (44.207)	167 (42.602)	81 (47.929)		
Smoking, n (%)				*χ*^2^= 0.523	0.469
No	366 (65.241)	252 (64.286)	114 (67.456)		
Yes	195 (34.759)	140 (35.714)	55 (32.544)		
Alcohol, n (%)				*χ*^2^= 0.004	0.947
No	364 (64.884)	254 (64.796)	110 (65.089)		
Yes	197 (35.116)	138 (35.204)	59 (34.911)		
Diabetes, n (%)				*χ*^2^= 0.023	0.880
No	513 (91.444)	358 (91.327)	155 (91.716)		
Yes	48 (8.556)	34 (8.673)	14 (8.284)		
Cardiopulmonary diseases, n (%)				*χ*^2^= 0.796	0.372
No	452 (80.570)	312 (79.592)	140 (82.840)		
Yes	109 (19.430)	80 (20.408)	29 (17.160)		
Distance from the anal verge, n (%)				*χ*^2^= 0.844	0.358
<5	259 (46.168)	176 (44.898)	83 (49.112)		
≥5	302 (53.832)	216 (55.102)	86 (50.888)		
TV, n (%)				*χ*^2^= 1.795	0.180
<7.51	352 (62.745)	253 (64.541)	99 (58.580)		
≥7.51	209 (37.255)	139 (35.459)	70 (41.420)		
Chemotherapy regimen				*χ*^2^= 0.009	0.924
combination	327 (58.289)	229 (58.418)	98 (57.988)		
capecitabine	234 (41.711)	163 (41.582)	71 (42.012)		
CEA, n (%)				*χ*^2^= 0.113	0.737
<3.85	238 (43.590)	167 (44.063)	71 (42.515)		
≥3.85	308 (56.410)	212 (55.937)	96 (57.485)		
CA199, n (%)				*χ*^2^= 0.009	0.922
<13.7	288 (54.340)	200 (54.201)	88 (54.658)		
≥13.7	242 (45.660)	169 (45.799)	73 (45.342)		
NEU, n (%)				*χ*^2^= 3.280	0.070
<5.24	466 (83.066)	333 (84.949)	133 (78.698)		
≥5.24	95 (16.934)	59 (15.051)	36 (21.302)		
WBC, n (%)				*χ*^2^= 1.287	0.257
<6.63	355 (63.280)	254 (64.796)	101 (59.763)		
≥6.63	206 (36.720)	138 (35.204)	68 (40.237)		
LYM, n (%)				*χ*^2^= 0.691	0.406
<1.54	207 (36.898)	149 (38.010)	58 (34.320)		
≥1.54	354 (63.102)	243 (61.990)	111 (65.680)		
HB, n (%)				*χ*^2^= 0.961	0.327
<137.5	313 (55.793)	224 (57.143)	89 (52.663)		
≥137.5	248 (44.207)	168 (42.857)	80 (47.337)		
ALB, n (%)				*χ*^2^= 3.135	0.077
<41.55	217 (38.681)	161 (41.071)	56 (33.136)		
≥41.55	344 (61.319)	231 (58.929)	113 (66.864)		
PLR, n (%)				*χ*^2^= 0.890	0.345
<174.4	206 (36.720)	139 (35.459)	67 (39.645)		
≥174.4	355 (63.280)	253 (64.541)	102 (60.355)		
NLR, n (%)				*χ*^2^= 0.258	0.611
<2.81	400 (71.301)	282 (71.939)	118 (69.822)		
≥2.81	161 (28.699)	110 (28.061)	51 (30.178)		
SII, n (%)				*χ*^2^= 0.015	0.903
<415	168 (29.947)	118 (30.102)	50 (29.586)		
≥415	393 (70.053)	274 (69.898)	119 (70.414)		
PNI, n (%)				*χ*^2^= 2.147	0.143
<48.08	139 (24.777)	104 (26.531)	35 (20.710)		
≥48.08	422 (75.223)	288 (73.469)	134 (79.290)		
LANR, n (%)				*χ*^2^= 0.252	0.616
<18.67	238 (42.424)	169 (43.112)	69 (40.828)		
≥18.67	323 (57.576)	223 (56.888)	100 (59.172)		

**Notes.**

*χ*^2^ Chi-square test

### Univariate and multivariate logistic regression analysis

**Figure 2:**
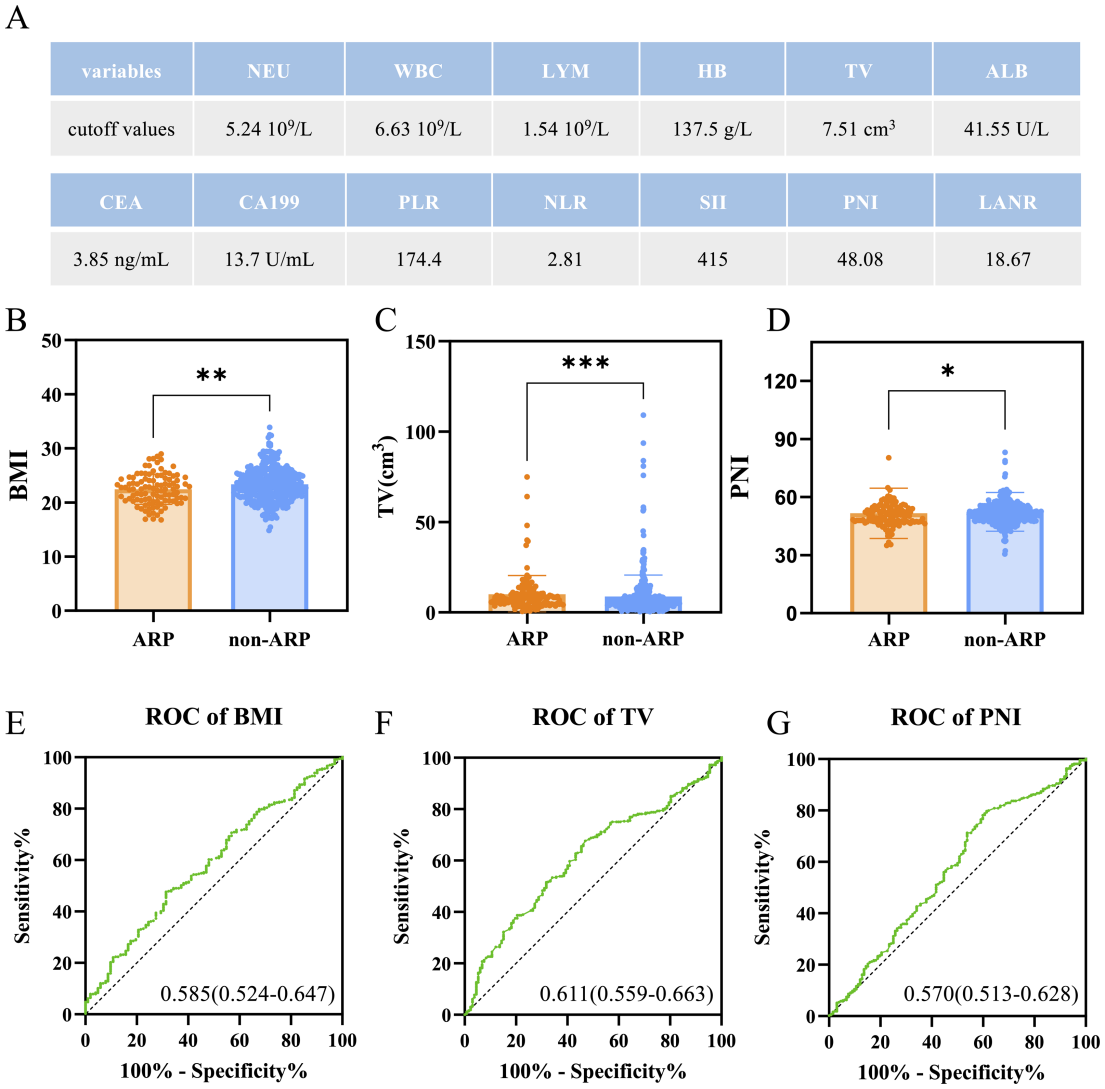
Distribution of key continuous variables and their ROC-based cutoff values for predicting ARP. (A) Cutoff values for hematologic and clinical variables determined by ROC analysis using the Youden index. (B–D) Comparison of BMI, tumor volume (TV), and prognostic nutritional index (PNI) between the ARP and non-ARP groups. Lower BMI and PNI, and higher TV were significantly associated with ARP. (E–G) ROC curves evaluating the predictive ability of BMI, TV, and PNI for ARP.

Cut-off values were determined using ROC curve analysis based on the Youden index (Fig. 2A). Univariable logistic regression analysis (Table 3) identified lower BMI, ALB, PNI, and high TV as predictors of ARP. Multivariable logistic regression identified lower BMI (<24), lower PNI (<48.08), and larger TV (>7.51 cm^3^) as independent predictors of ARP, with OR and 95% CI as follows: (0.394, 0.230−0.675, *P* < 0.001), (0.470, 0.279−0.790, *P* = 0.004), (2.242, 1.366−3.680, *P* = 0.001), respectively. Each predictor had a variance inflation factor (VIF) below 10 and a tolerance above 0.1, indicating an absence of multicollinearity. Chemotherapy regimen was included in the multivariable model but did not reach statistical significance. The number of chemotherapy cycles was not entered into the main model because this variable applies only to patients receiving combination chemotherapy. In a sensitivity analysis restricted to combination-therapy patients, the number of cycles did not materially change the associations between BMI, TV, or PNI and ARP.

**Table 3:** Univariate and Multivariate analysis of parameters associated with ARP.

Variables	**Univariate analysis**		**Multivariate analysis**	
	*P*	OR (95% CI)	*P*	OR (95% CI)
Gender				
Female *vs.* Male (Ref)	0.318	0.783 (0.484 ∼1.266)		
Age				
≥ 60 vs.<60 (Ref)	0.361	0.805 (0.505 ∼1.283)		
BMI				
≥ 24 *vs.*<24 (Ref)	**<**.**001**	0.364 (0.217 ∼0.610)	**<**.**001**	0.394 (0.230 ∼0.675)
Smoking				
No *vs.* Yes (Ref)	0.090	0.647 (0.391 ∼1.070)		
Alcohol				
No *vs.* Yes (Ref)	0.274	0.758 (0.461 ∼1.245)		
Diabetes				
No *vs.* Yes (Ref)	0.750	1.139 (0.512 ∼2.533)		
Cardiopulmonary diseases				
No *vs.* Yes (Ref)	0.486	0.810 (0.447 ∼1.466)		
Distance from the anal verge				
≥ 5 vs.<5 (Ref)	0.877	1.037 (0.652 ∼1.651)		
TV				
≥ 7.51 *vs.*<7.51 (Ref)	**<**.**001**	2.450 (1.528 ∼3.929)	**0**.**001**	2.242 (1.366 ∼3.680)
Chemotherapy regimen				
Capecitabine *vs.* Combination (Ref)	0.121	1.463 (0.905 ∼2.365)		
CEA				
≥3.85 *vs.*<3.85 (Ref)	0.302	0.567 (0.192 ∼1.668)		
CA199				
≥ 13.7 *vs.*<13.7 (Ref)	0.160	0.706 (0.435 ∼1.148)		
NEU				
≥ 5.24 *vs.*<5.24 (Ref)	0.279	0.679 (0.337 ∼1.368)		
WBC				
≥6.63 *vs.*<6.63 (Ref)	0.722	0.915 (0.562 ∼1.490)		
LYM				
≥1.54 *vs.*<1.54 (Ref)	0.236	0.753 (0.470 ∼1.204)		
HB				
≥137.5 *vs.*<137.5 (Ref)	0.262	0.763 (0.475 ∼1.225)		
ALB				
≥ 41.55 *vs.*<41.55 (Ref)	**0**.**017**	0.568 (0.357 ∼0.906)		
PLR				
≥174.4 *vs.*<174.4 (Ref)	0.938	0.981 (0.606 ∼1.589)		
NLR				
≥2.81 *vs.*<2.81 (Ref)	0.725	1.096 (0.659 ∼1.823)		
SII				
≥415 *vs.*<415 (Ref)	0.918	0.974 (0.589 ∼1.609)		
PNI				
≥48.08 *vs.*<48.08 (Ref)	**<**.**001**	0.429 (0.262 ∼0.702)	**0**.**004**	0.470 (0.279 ∼0.790)
LANR				
≥18.67 *vs.*<18.67 (Ref)	0.231	0.753 (0.473 ∼1.197)		

**Notes.**

Bold values indicate statistical significance (*P* < 0.05).

### The association between BMI, TV, PNI and ARP

Utilizing multivariate analysis, we evaluated the differences in BMI, TV, and PNI as continuous variables between the non-ARP and ARP groups, and analyzed their predictive capabilities for ARP (Figs. 2B–2D). It was in accordance with the logistic regression analysis. Patients in the ARP group had lower BMI (23.31 *vs.* 22.48, *P* = 0.009), PNI (51.65 *vs.* 50.43, *p* = 0.014), and higher TV (5.560 *vs.* 7.712, *p* < 0.001) compared with non-ARP group. Furthermore, the predictive ability of BMI, TV, and PNI was calculated by ROC curve, which AUC (95% CI) values were 0.585 (0.524−0.647), 0.611 (0.559−0.663), and 0.570 (0.513−0.628), respectively (Figs. 2E–2G). It indicates that BMI, PNI and TV alone as predictors had low predictive power for ARP occurrence.

### Construction and validation of the nomogram

**Figure 3:**
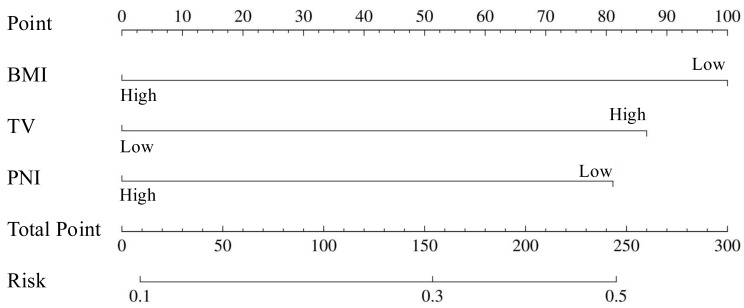
Nomogram predicting the risk of ARP. The nomogram integrates three independent predictors—BMI, TV, and PNI. Each variable corresponds to a point value, and the sum of these points aligns with a predicted probability of ARP. Lower BMI (<24 kg/m^2^), higher TV (>7.51 cm^3^), and lower PNI (<48.08) contribute to higher risk scores.

**Figure 4:**
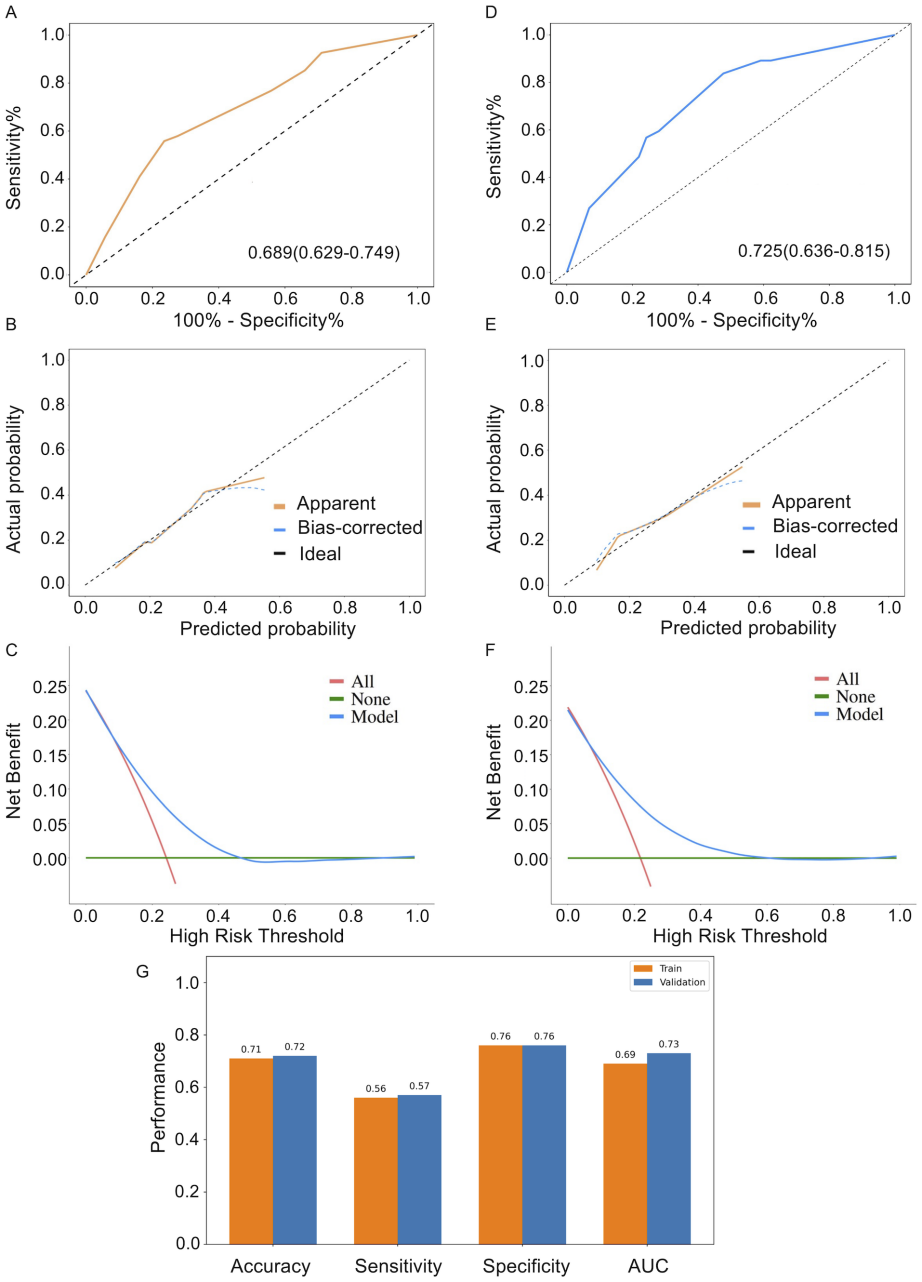
Model performance of the ARP prediction nomogram in the training and validation cohorts. (A) ROC curve of the nomogram in the training cohort showing moderate discrimination (AUC = 0.689). (B) Calibration curve demonstrating good agreement between predicted and observed ARP risk in the training cohort. (C) Decision curve analysis (DCA) indicating net clinical benefit of the nomogram across relevant threshold probabilities. (D) ROC curve of the nomogram in the validation cohort (AUC = 0.725), confirming external predictive performance. (E) Calibration curve in the validation cohort showing appropriate model fit. (F) DCA in the validation cohort supporting clinical utility. (G) Comparison of model accuracy, sensitivity, specificity, and AUC between training and validation cohorts.

To develop a multivariable prediction model, a nomogram model based on BMI, TV, and PNI was constructed (Fig. 3). The final multivariable logistic regression model identified BMI < 24 kg/m^2^, TV > 7.51 cm^3^, and PNI < 48.08 as independent predictors of acute radiation proctitis. The model coefficients, including the intercept and beta estimates for each predictor, are provided in Table S2. These coefficients allow complete numerical reconstruction of the nomogram. Variables that remained significant in the final model included BMI, TV and PNI. The final model equation was:

logit(P) = −0.5947 − 0.9315  × BMI + 0.8073  × TV − 0.7554  × PNI,

where BMI ≥ 24 kg/m^2^, TV > 7.51 cm^3^, and PNI ≥ 48.08 are binary variables coded as 1 if the corresponding condition is met and 0 otherwise, and P denotes the probability of ARP. In the training cohort, the nomogram demonstrates superior AUC performance (AUC: 0.689; 95% CI [0.629–0.749]) in identifying ARP compared to BMI, PNI, and TV individually (Fig. 4A). To provide an example of potential clinical application, a predicted-probability threshold of 0.332 was examined in the validation cohort. At this threshold, the model yielded a sensitivity of 0.568, specificity of 0.758, positive predictive value (PPV) of 0.396, and negative predictive value (NPV) of 0.862. The calibration curves demonstrated satisfactory alignment between ARP predictions and actual observations, with the Hosmer-Lemeshow test confirming an adequate fit for the nomogram (*P* > 0.05) (Fig. 4B). DCA demonstrated significant positive net benefits at the threshold probabilities (Fig. 4C). Furthermore, results from the validation cohort also support that observation. The nomogram’s AUC (0.725; 95% CI [0.636–0.815]) was higher than any individual predictor (Fig. 4D). Calibration curves and DCA validated the nomogram’s accuracy and clinical applicability (Figs. 4E–4F). To enhance internal validity, bootstrap resampling with 500 repetitions was performed according to TRIPOD recommendations. The apparent AUC of the model was 0.703, and the optimism-corrected AUC was 0.698. The calibration intercept and slope were 0.04 and 1.04 (after correction: 0.11 and 1.07), indicating good overall calibration.

### The relationship between clinicopathologic features, hematological parameters and PNI

**Figure 5:**
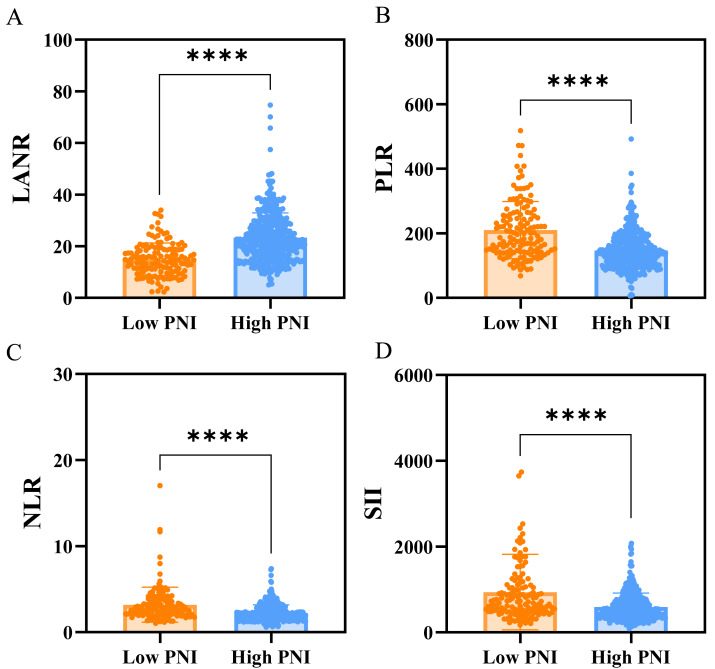
Relationship between PNI level and inflammation-related indices. (A) LANR was significantly lower in the low-PNI group. (B–D) Markers of systemic inflammation (PLR, NLR, and SII) were significantly higher among patients with low PNI, suggesting that poorer nutritional status is associated with enhanced systemic inflammatory response.

Of the 561 patients studied, 422 (75.22%) were classified as high PNI, and 139 (24.78%) as low PNI. The analysis of clinicopathologic features and hematological parameters indicated that the low PNI group had a greater proportion of patients with lower BMI, HB, ALB, LANR, and higher TV compared to the high PNI group. Details were shown in Table 4. Analysis indicated that patients with lower PNI exhibited reduced LANR levels (Fig. 5A). In addition, poorer nutritional status was associated with higher inflammation markers. Patients with lower PNI exhibited elevated PLR, NLR, and SII levels compared to those with higher PNI (Figs. 5B–5D).

**Table 4:** The association between clinical and pathological characteristics and PNI.

Variables	Total (*n* = 561)	Low PNI (*n* = 139)	High PNI (*n* = 422)	Statistic	*P*
Gender, n (%)				*χ*^2^= 0.000	0.987
Female	190 (33.868)	47 (33.813)	143 (33.886)		
Male	371 (66.132)	92 (66.187)	279 (66.114)		
Age, n (%)				*χ*^2^= 1.090	0.296
<60	316 (56.328)	73 (52.518)	243 (57.583)		
≥ 60	245 (43.672)	66 (47.482)	179 (42.417)		
BMI, n (%)				*χ*^2^= 9.253	**0**.**002**
<24	313 (55.793)	93 (66.906)	220 (52.133)		
≥ 24	248 (44.207)	46 (33.094)	202 (47.867)		
Smoking, n (%)				*χ*^2^= 0.073	0.787
No	366 (65.241)	92 (66.187)	274 (64.929)		
Yes	195 (34.759)	47 (33.813)	148 (35.071)		
Alcohol, n (%)				*χ*^2^= 1.947	0.163
No	364 (64.884)	97 (69.784)	267 (63.270)		
Yes	197 (35.116)	42 (30.216)	155 (36.730)		
Diabetes, n (%)				*χ*^2^= 0.001	0.970
No	513 (91.444)	127 (91.367)	386 (91.469)		
Yes	48 (8.556)	12 (8.633)	36 (8.531)		
Cardiopulmonary diseases, n (%)				*χ*^2^= 1.532	0.216
No	452 (80.570)	117 (84.173)	335 (79.384)		
Yes	109 (19.430)	22 (15.827)	87 (20.616)		
Distance from the anal verge, n (%)				*χ*^2^= 0.128	0.720
<5	259 (46.168)	66 (47.482)	193 (45.735)		
≥ 5	302 (53.832)	73 (52.518)	229 (54.265)		
TV, n (%)				*χ*^2^= 15.107	**<0.001**
<7.51	352 (62.745)	68 (48.921)	284 (67.299)		
≥ 7.51	209 (37.255)	71 (51.079)	138 (32.701)		
CEA, n (%)				*χ*^2^= 1.111	0.292
<3.85	238 (43.590)	54 (39.706)	184 (44.878)		
≥ 3.85	308 (56.410)	82 (60.294)	226 (55.122)		
CA199, n (%)				*χ*^2^= 3.082	0.079
<13.7	288 (54.340)	81 (60.902)	207 (52.141)		
≥ 13.7	242 (45.660)	52 (39.098)	190 (47.859)		
NEU, n (%)				*χ*^2^= 0.014	0.904
<5.24	466 (83.066)	115 (82.734)	351 (83.175)		
≥ 5.24	95 (16.934)	24 (17.266)	71 (16.825)		
WBC, n (%)				*χ*^2^= 1.046	0.306
<6.63	355 (63.280)	93 (66.906)	262 (62.085)		
≥ 6.63	206 (36.720)	46 (33.094)	160 (37.915)		
LYM, n (%)				*χ*^2^= 68.079	**<0.001**
<1.54	207 (36.898)	92 (66.187)	115 (27.251)		
≥ 1.54	354 (63.102)	47 (33.813)	307 (72.749)		
HB, n (%)				*χ*^2^= 38.347	**<0.001**
<137.5	313 (55.793)	109 (78.417)	204 (48.341)		
≥ 137.5	248 (44.207)	30 (21.583)	218 (51.659)		
ALB, n (%)				*χ*^2^= 222.200	**<0.001**
<41.55	217 (38.681)	128 (92.086)	89 (21.090)		
≥ 41.55	344 (61.319)	11 (7.914)	333 (78.910)		
PLR, n (%)				*χ*^2^= 34.713	**<0.001**
<174.4	206 (36.720)	22 (15.827)	184 (43.602)		
≥ 174.4	355 (63.280)	117 (84.173)	238 (56.398)		
NLR, n (%)				*χ*^2^= 29.466	**<0.001**
<2.81	400 (71.301)	74 (53.237)	326 (77.251)		
≥ 2.81	161 (28.699)	65 (46.763)	96 (22.749)		
SII, n (%)				*χ*^2^= 15.816	**<0.001**
<415	168 (29.947)	23 (16.547)	145 (34.360)		
≥ 415	393 (70.053)	116 (83.453)	277 (65.640)		
LANR, n (%)				*χ*^2^= 75.908	**<0.001**
<18.67	238 (42.424)	103 (74.101)	135 (31.991)		
≥ 18.67	323 (57.576)	36 (25.899)	287 (68.009)		

**Notes.**

*χ*^2^ Chi-square test

Bold values indicate statistical significance (*P* < 0.05).

## Discussion

In this study, clinical characteristics, inflammation and nutritional indices and their related scores were evaluated in LARC patients undergoing nCRT, and their relationships with ARP results were examined. TV, BMI, ALB and PNI were found to be the predictors of ARP. Although chemotherapy regimen was adjusted for in the multivariable analysis, it was not a significant predictor of ARP. The number of chemotherapy cycles is regimen-specific and therefore was not included in the primary model. A sensitivity analysis limited to patients receiving combination chemotherapy showed that cycle number did not significantly affect the main findings, suggesting that residual confounding from chemotherapy intensity is unlikely to change the overall conclusions. When the TV is large, a higher dose and a broader irradiation range are required during radiotherapy to control the tumor. This increases the volume and dose of rectal exposure, thereby increasing the risk of radiation proctitis (Rijkmans et al., 2019).

Tumors contribute to reduced adipose tissue accumulation, leading to protein-energy malnutrition (Porporato, 2016). BMI and ALB levels were all likely associated with nutritional status. Our study identified lower BMI as a significant risk factor for ARP. BMI represents weight loss, which is characterized by a decrease in adipose tissue and muscular tissue. Therefore, a lower BMI could lead to increased radiation dose concentration in the pelvic region, heightening local tissue damage and the risk of ARP (Liu et al., 2021a). The study identified lower serum ALB levels as a high-risk factor for ARP, typically indicating malnutrition(Li et al., 2022). Protein-calorie malnutrition is linked to increase in patient mortality risk in radiation proctitis cases (Dahiya et al., 2022; Gangadharan et al., 2017; Marshall, 2016). Additionally, inadequate nutrition impairs patients’ immune function, diminishing their capacity to handle radiation-induced inflammation and extending tissue damage (Matei, Winters-Stone & Raber, 2023). Nutritional status is strongly related to inflammaging. Malnutrition can extend the inflammatory phase by inhibiting fibroblast proliferation, collagen formation, tensile strength, and angiogenesis, thereby adversely impacting wound healing (Stechmiller, 2010).

Unlike BMI and ALB, PNI provides a comprehensive assessment of both inflammatory and nutritional status. It originates from serum albumin and lymphocyte count, both well-established indicators of nutritional status and inflammation. Among the inflammation and nutrition scores (NLR, PLR, SII, PNI, LANR) examined in this study, only the PNI was found as the predictor for ARP. Elevated PNI levels correlate with improved prognosis in various cancers (Haraga et al., 2016; Jin et al., 2018; Li et al., 2018). Numerous studies have indicated that a lower pre-treatment PNI correlates with a poorer prognosis in RC patients (Pian & Oh, 2024; Qiu et al., 2024; Silva et al., 2024). A prior study indicated that a low PNI correlates with severe acute adverse events of any kind in head and neck patients undergoing radical or adjuvant radiotherapy (Kono et al., 2017), without specifying individual toxicity types. Another study indicated that head and neck cancer (HNC) patients with low PNI undergoing chemoradiotherapy experienced increased incidences of feeding tube placement, grade 3–4 hematological toxicities, and sepsis (Chang et al., 2018). This suggests the potential of low PNI values to predict high radiotherapy toxicity. To date, no studies have examined the impact of PNI on ARP in rectal cancer patients. Furthermore, we found that low PNI values are strongly associated with high inflammation-related scores, which further confirms the notion that malnutrition may exacerbate inflammatory responses.

Although TV, BMI, and PNI are all independent predictors of ARP (Dalsania et al., 2021; Li et al., 2018), their predictive capabilities are limited. The nomogram constructed using these three indicators has a better ability to screen patients with a high likelihood of developing ARP. Although the discriminative performance of the model was modest, it may still have potential clinical utility as a risk stratification tool. Rather than serving as a stand-alone decision-making instrument, the model could help identify patients at relatively higher risk of ARP who may benefit from closer monitoring or early supportive interventions. Recent advances in artificial intelligence (AI) have introduced machine-learning and deep-learning models for cancer diagnosis, treatment response prediction, and radiotherapy toxicity assessment (Alaca, 2025; Wang et al., 2021b). These AI-based models can integrate imaging, dosimetric, and clinical variables to capture complex nonlinear patterns that may improve predictive accuracy (Poirion et al., 2021). However, their implementation typically requires large, annotated datasets and high computational resources, limiting their use in routine clinical practice. In contrast, our nomogram provides a simple and interpretable tool based on readily available clinical indicators, offering a practical alternative for ARP risk stratification in real-world settings. Nutrition support therapy is essential throughout the entire treatment process for these patients. The present study has several strengths. This study’s findings may extend the clinical application of PNI by providing a cut-off value for patient stratification, aligning with existing research that suggests values between 40 and 52 (Fanetti et al., 2021), with our cut-off at 48.08. Conversely, radiation techniques are continuously evolving into advanced treatment modalities with reduced toxicity. Our study focused on patients treated with IMRT and VMAT, techniques that minimize exposure of organs at risk (OARs) to radiotherapy, thereby reducing toxicity. We focused exclusively on rectal cancer patients receiving nCRT, and our findings suggest that the nomogram may play a role in predicting ARP.

This study has several limitations. One important limitation is that detailed radiotherapy dosimetric parameters were not available and therefore could not be included in the analysis. As a result, potential confounding related to radiation dose distribution could not be fully accounted for. In addition, PNI was calculated from a single blood sample and may therefore be influenced by transient fluctuations. Finally, this was a retrospective single-center study, and the nomogram has not yet undergone external validation. Future multi-center studies with independent cohorts are needed to validate and further refine the model.

##  Supplemental Information

10.7717/peerj.21364/supp-1 Supplemental Information 1 Univariate and Multivariate analysis of parameters associated with ARP in patients treated with CAPOX/FOLFOX

10.7717/peerj.21364/supp-2 Supplemental Information 2 The intercept and beta coefficients for each predictor

10.7717/peerj.21364/supp-3 Supplemental Information 3 Anonymized dataset for ARP prediction in LARC patients undergoing nCRT

10.7717/peerj.21364/supp-4 Supplemental Information 4 Data dictionary for ARP prediction in LARC patients undergoing nCRT

## Additional Information and Declarations

### Data Availability

The following information was supplied regarding data availability: The raw data is available in the Supplemental File.
