# Supplementary material for: Nomogram based on Prognostic Nutritional Index (PNI) for predicting acute radiation proctitis in locally advanced rectal cancer patients with neoadjuvant chemoradiotherapy
Source: PeerJ. 2026 Jun 2;14:e21364. doi: 10.7717/peerj.21364 (PMC13239477; doi:10.7717/peerj.21364)
Supplement: Supplemental Information 1 [file peerj-14-21364-s001.docx]

**Supplementary Table 1 Univariate and Multivariate analysis of parameters associated with ARP in patients treated with CAPOX/FOLFOX**

| Variables | **Univariate analysis** | | **Multivariate analysis** | |
| --- | --- | --- | --- | --- |
|  | *P* | OR (95%CI) | *P* | OR (95%CI) |
| Gender |  |  |  |  |
| Female (Ref) vs. Male | 0.986 | 1.006 (0.521 ~ 1.941) |  |  |
| Age |  |  |  |  |
| ≥ 60 vs.＜60(Ref) | 0.119 | 1.638 (0.882 ~ 3.043) |  |  |
| BMI |  |  |  |  |
| ≥ 24 vs.＜24(Ref) | **0.002** | 0.332 (0.166 ~ 0.663) | **0.008** | 0.376 (0.183 ~ 0.774) |
| Smoking |  |  |  |  |
| No (Ref) vs.Yes | 0.629 | 0.851 (0.443 ~ 1.636) |  |  |
| Alcohol |  |  |  |  |
| No (Ref) vs.Yes | 0.791 | 1.090 (0.578 ~ 2.054) |  |  |
| Diabetes |  |  |  |  |
| No (Ref) vs.Yes | 0.474 | 0.570 (0.122 ~ 2.655) |  |  |
| Cardiopulmonary diseases |  |  |  |  |
| No (Ref) vs.Yes | 0.244 | 1.599 (0.726 ~ 3.523) |  |  |
| Distance from the  anal verge |  |  |  |  |
| ≥ 5 vs.＜5(Ref) | 0.499 | 1.238 (0.666 ~ 2.300) |  |  |
| TV |  |  |  |  |
| ≥ 7.51 vs.＜7.51(Ref) | **<.001** | 2.916 (1.558 ~ 5.459) | **0.007** | 2.471 (1.279 ~ 4.775) |
| Chemotherapy cycles |  |  |  |  |
| ≥3 vs.＜3(Ref) | 0.165 | 2.011 (0.749 ~ 5.395) |  |  |
| CEA |  |  |  |  |
| ≥3.85 vs.＜3.85(Ref) | 0.854 | 1.060 (0.568 ~ 1.981) |  |  |
| CA199 |  |  |  |  |
| ≥ 13.7vs.＜13.7(Ref) | 0.066 | 0.533 (0.273 ~ 1.042) |  |  |
| NEU |  |  |  |  |
| ≥ 5.24 vs.＜5.24(Ref) | 0.060 | 0.307 (0.089 ~ 1.052) |  |  |
| WBC |  |  |  |  |
| ≥6.63 vs.＜6.63(Ref) | 0.260 | 0.692 (0.364 ~ 1.313) |  |  |
| LYM |  |  |  |  |
| ≥1.54 vs.＜1.54(Ref) | 0.119 | 0.611 (0.329 ~ 1.134) |  |  |
| HB |  |  |  |  |
| ≥137.5 vs.＜137.5(Ref) | 0.342 | 0.742 (0.401 ~ 1.373) |  |  |
| ALB |  |  |  |  |
| ≥ 41.55 vs.＜41.55(Ref) | **0.008** | 0.429 (0.230 ~ 0.800) |  |  |
| PLR |  |  |  |  |
| ≥174.4vs.＜174.4(Ref) | 0.576 | 1.205 (0.627 ~ 2.314) |  |  |
| NLR |  |  |  |  |
| ≥2.81 vs.＜2.81(Ref) | 0.765 | 0.876 (0.367 ~ 2.090) |  |  |
| SII |  |  |  |  |
| ≥415 vs.＜415(Ref) | 0.925 | 0.967 (0.481 ~ 1.944) |  |  |
| PNI |  |  |  |  |
| ≥48.08 vs.＜48.08(Ref) | **<.001** | 0.271 (0.140 ~ 0.526) | **0.001** | 0.322 (0.161 ~ 0.642) |
| LANR |  |  |  |  |
| ≥18.67 vs.＜18.67(Ref) | 0.234 | 0.689 (0.373 ~ 1.273) |  |  |
