# Supplementary material for: Nomogram based on Prognostic Nutritional Index (PNI) for predicting acute radiation proctitis in locally advanced rectal cancer patients with neoadjuvant chemoradiotherapy
Source: PeerJ. 2026 Jun 2;14:e21364. doi: 10.7717/peerj.21364 (PMC13239477; doi:10.7717/peerj.21364)
Supplement: Supplemental Information 2 [file peerj-14-21364-s002.docx]

| Predictor | β coefficient | Standard Error | Odds Ratio (95% CI) |
| --- | --- | --- | --- |
| Intercept | −0.5947 | 0.2581 | — |
| BMI (<24 vs ≥24 kg/m²) | −0.9315 | 0.2746 | 0.394 (0.230–0.675) |
| TV (>7.51 vs ≤7.51 cm³) | +0.8073 | 0.2528 | 2.242 (1.366–3.680) |
| PNI (<48.08 vs ≥48.08) | −0.7554 | 0.2652 | 0.470 (0.279–0.790) |

**Supplementary table 2 The intercept and beta coefficients for each predictor**
